# Supplementary material for: Impact of the Gestational Diabetes Diagnostic Criteria during the Pandemic: An Observational Study
Source: J Clin Med. 2021 Oct 24;10(21):4904. doi: 10.3390/jcm10214904 (PMC8585066; doi:10.3390/jcm10214904)
Supplement: Supplementary file 1 [file jcm-10-04904-s001.zip › jcm-1394981-supplementary.pdf]

Table S1. Comparison between women in 2019-group vs. 2020-group: GDM risk factors, obstetrical and perinatal outcomes.

	2019-group (n=237)	2020-group (n=255)	p
**GDM riskfactors**			
Age (years)	33.3±5.2	33.8±5.0	0.214
BMI before pregnancy (kg/m^2)	27.8±6.7	27.1±6.1	0.316
Nulligravidwomen	112 (47.2%)	107 (41.9%)	**0.013**
Familyhistory of diabetes	59 (24.9%)	79 (30.9%)	0.447
GDM in a prior pregnancy	11 (4.6%)	29 (11.4%)	**0.013**
Previousmacrosomia	5 (2.1%)	26 (10.2%)	**0.001**
**Obstetrical data**			
Maternal weightgain (Kg)	9.94±5.23	10.29±5.87[a]	0.571
BMI prepartum (kg/m^2)	31.35± 5.99	30.71± 5.71	0.333
Hypertensive disorders of pregnancy [a]	13 (5.5 %)	18 (7.1%)	0.462
Gestationalweek at birth	38.90 ±1.77	38.76 ±2.30	0.485
Type of delivery			
• Non-instrumental	123 (51.9 %)	117 (45.9 %)	
• Instrumental	24 (10.1 %)	35 (13.7 %)	0.300
• Cesarea	63 (26.6 %)	58 (22.7 %)	
**Perinatal data**			
Pretermbirth [b]	15 (7.0 %)	19 (8.7 %)	0.095
Birthweight (g)	3269 ± 552	3333 ± 564	0.247
Birthlength (cm)	49.95±2.30	50.48±2.62	**0.038**
Birthweight pc	54.86±29.96	57.72±30.23	0.341
Birthweight>90th pc	33 (13.9 %)	40 (15.7 %)	0.476
Birthweight<3rd pc	6 (2.5 %)	4 (1.6 %)	
**Perinatal complications**			
Any perinatal complication	28 (11.8 %)	23 (9.0 %)	0.310
• Neonatal hypoglycemia	4 (1.7%)	5 (2%)	0.821
• Jaundicerequiringphototherapy	12 (5.1%)	11 (4.3%)	0.694
• Hospitalization	16 (6.8%)	11 (4.3%)	0.236
• Respiratorydistresssyndrome	4 (1.7%)	8 (3.1%)	0.298
• NICUadmission	7 (3%)	4 (1.6%)	0.299
• Perinatal death	2 (0.8%)	2 (0.8%)	0.941
• Obstetric trauma	2 (0.8%)	1 (0.4%)	0.520

GDM: gestational diabetes mellitus; pc: percentile; NICU: Neonatal IntensiveCareUnit. [a].Includes: pre-pregnancyhypertension, gestationalhypertension, preeclampsia. [b].Gestational age at birth ≤ 36 weeks.
